# Pesticide Residues and Health Risk Assessment in Tomatoes and Lettuces from Farms of Metropolitan Region Chile

**DOI:** 10.3390/molecules25020355

**Published:** 2020-01-15

**Authors:** Sebastian Elgueta, Marcela Valenzuela, Marcela Fuentes, Pablo Meza, Juan Pablo Manzur, Shaofeng Liu, Guoqing Zhao, Arturo Correa

**Affiliations:** 1 Laboratory of Pesticide Residues and Environment, Instituto de Investigaciones Agropecuarias, INIA Centro Regional La Platina, Santiago 8820000, Chile; marcela.valenzuela@inia.cl (M.V.); mfuentes@inia.cl (M.F.); acorrea@inia.cl (A.C.); 2 Núcleo de Investigaciones Aplicadas en Ciencias Veterinarias y Agronómicas, Universidad de las Américas, Sede Providencia, Santiago 7500000, Chile; 3 Department of Plant Health and Vegetables, Instituto de Investigaciones Agropecuarias, INIA Centro Regional La Platina, Santiago 8820000, Chile; pablo.meza@inia.cl (P.M.); jpmanzur@inia.cl (J.P.M.); 4 Plymouth Business School, University of Plymouth, Drake Circus, Plymouth PL4 8AA, UK; shaofeng.liu@plymouth.ac.uk (S.L.); guoqing.zhao@plymouth.ac.uk (G.Z.)

**Keywords:** methamidophos, methomyl, health risk assessment, Metropolitana Region of Chile, Maximum Residue Levels (MRL), Hazard Quotient HQ

## Abstract

Over the last years, the detection of pesticide residues in the official food surveillance programs of Chile has been increased, mainly in fresh vegetables such as tomatoes and lettuces. The Metropolitana Region of Chile presents the highest detections in the country. The lack of evaluations of toxicological risks in human health have increased uncertainty of the potential effects of pesticides exposures in the Chilean population. This research aims to determinate health risks assessment of pesticide residues associated to tomatoes and lettuces produced in Metropolitana Region. The findings of this study reveal that tomatoes and lettuces cultivated in the MR show more than 50% of samples with one or multiple pesticides residues. From the total samples, 16% were over the Chilean Maximum Residue Levels (MRLs). The main pesticides detected in tomatoes and lettuces were methamidophos, methomyl, difenoconazole, cyprodinil and boscalid. The results obtained using the official data of the Ministry of Health of Chile (MINSAL) compared to the World Health Organization (WHO), describe relevant risks through the Estimated Daily Intakes (EDI), Hazard Quotients (HQ) and Hazard Index (HI) for the Chilean population due to high concentrations of methamidophos, methomyl and cyprodinil. More restrictions for the use of methamidophos, methomyl, difenoconazole, cyprodinil and boscalid and effective control programs should be implemented in order to mitigate the impacts on the Chilean population.

## 1. Introduction

In the conventional cultivation of fresh vegetables, pesticides are used during the vegetation period to increase their yield, prior to production or as post-harvest treatment [1]. Different treatments with multiple pesticides are necessary to ensure a good production and reduce the impact of crop diseases [2]. During the applications of pesticides in farms, factors such as frequency application, equipment, mixer conditions and exposure determine the potential chemical risks to human health [3]. The consumption of fresh vegetables can be considered as the primary route of exposure to pesticides through ingestion in human diet, which accounts for 20–40% of the total food consumption [4,5,6]. The pesticide residues in fresh vegetables have been monitored for decades in most developed countries. However, they are not properly reported in developing countries [7]. Surveillance programs and enforcement systems are recognized as relevant initiatives to reduce potential hazards of pesticides to human health [8]. The official surveillance programs in different countries have been focused on testing pesticide levels to improve food safety and agricultural practices as well as to minimize economic losses [9].

The National Pesticide Residues Monitoring Program (NPRMP) in Chile has been focusing on the proper use of pesticides concerning application rates and compliance with MRLs both for their authorization and registration [10]. In Chile, the extensive use of pesticides in farms has led to many difficulties due to the high presence of residues after harvest, thus becoming one of the most important issues in the area of chemical food safety [11,12]. The country has one of the most significant pesticide markets in Latin America, with over 500 active ingredients and more than 1000 formulations registered for different crops [13]. The highest levels of pesticide use may contribute to a greater health risk; hence, a detailed pesticide residue analysis and risk assessment are necessary [9].

For 2019, the NPRMP shows a critical situation in terms of the high number of transgressions of Chilean MRLs, mainly in fresh vegetables for pesticides such as methamidophos, chlorpyrifos, methomyl and γ-cyhalothrin [11,12]. Methamidophos ((RS)-(*O*,*S*-dimethyl phosphoramidothioate)) is hydrophilic with a high water solubility often transported to vegetables. This insecticide can be transferred to plants from the cultivation medium through root uptake and translocation, and its associated risk is higher for fresh vegetables that may be consumed raw [14]. Many studies have demonstrated the acute toxic effects of methamidophos by inhibiting acetylcholinesterase and thus be hazardous to human health [15,16]. A similar situation has been described for methomyl (*S*-methyl (EZ)-*N*-(methylcarbamoyloxy) thioacetimidate). This insecticide is a carbamate that is highly soluble in water (5.8 g/100 g) and very toxic. The US Environmental Protection Agency (EPA) classified Methomyl as a Class I restricted-use pesticide [17]. Both pesticides are the most frequently found in the monitoring program, mainly in the Metropolitana Region of Chile [11,12]. The cumulative exposure considers the possibility of simultaneous exposure to a group of compounds that have a common mechanism or mode of action [18]. A preliminary risk assessment in Chile revealed a potential risk posed by the consumption of leafy vegetables with high concentrations of methamidophos from north-central agricultural areas of Chile [19]. We recently reported the pesticide residues detected in ready-to-eat leafy vegetables commercialized in Santiago, Chile. The main conclusion presented in this study explains the health risks associated with the high residual concentrations of methamidophos and chlorpyrifos [20].

In Chilean agriculture, relevant problems of pesticide residues have been detected in the national surveillance program, mainly in vegetables such as lettuce, tomatoes, peppers, chard, cucumbers and spinach [11,12]. The lettuces and tomatoes represent the most important production of vegetables in Chile with 6518 and 5269 ha cultivated in the country in 2017, and their consumption is increasing every year with 50 and 120 gr/day respectively [21,22]. These vegetables are commonly grown in winter and summer season usually are highly exposed to pesticides due to overuse or wrong application by farmers. Most of fresh vegetables distributed in Chile are commercialized in Lo Valledor, located in the Metropolitana Region, which is the major center of distribution of vegetables and fruit. The food distributed in Lo Valledor can be obtained from all regions of Chile and their traceability and origin is unknown. Due to the increasing detections of organophosphates, carbamates and neonicotinoids mainly in the Metropolitana Region, there is a need to evaluate their impacts on human health. Therefore, the aim of this study was to evaluate pesticide residues and their associated health risk assessment in tomatoes and lettuces obtained from local farms in the most relevant region of Chile.

## 2. Results and Discussion

### 2.1. Quality Assurance of Method

All pesticide residues detected in tomatoes and lettuces were analysed according to the guidance document on analytical quality control and method validation procedures for pesticides residues and analysis in food and feed (SANTE) [23]. The accuracy and precision were expressed as the recoveries and relative standards deviations at different levels, respectively (Table 1). The recovery rates and the relative standard deviation (RSD) values ranged between 72–116% and 1–14%, respectively.

The limit of detection (LODs) were estimated between 5–10 µg/kg and the limit of quantification (LOQs) between 10–20 µg/kg according the guideless of validations and reproducibility [23]. Similar results have been reported with recoveries from 89–105%, RSD between 1.5–3.9%, LODs between 5–20 ng/mL and LOQs between 8–30 ng/mL for the analysis of organophosphorus pesticides in different vegetables including tomatoes [24].

### 2.2. Pesticide Residues Concentrations

In the determination of organochlorines and organophosphates pesticides in tomatoes, several parameters have been reported including LODs from 0.4 to 3.0 µg/kg, LOQs between 0.5–3.5 µg/kg and recoveries between 76% to 91% [25]. Results reported for tomatoes and other vegetables showed recoveries between 73% to 115%, RSD values lower than 20%, and LODs and LOQs lower than 0.01 mg/kg, for almost 200 of the analysed pesticides [26]. During the year 2018, 80 samples of tomatoes and lettuces obtained from local farms of Metropolitana Region were analysed to determine pesticide residues and their compliance with the Chilean MRLs.

Different substances, including insecticides and fungicides, were the main pesticide types. The screening in tomatoes and lettuces revealed several pesticides such as imidacloprid, γ-cyhalothrin, methamidophos, chlorpyrifos, dithiocarbamates, propamocarb, difenoconazole, azoxystrobin among others. Supporting our findings, the national surveillance program of Chile conducted by SAG reported similar pesticides for both vegetables in the Metropolitana Region during the year 2016 and 2017 [11,12].

In literature, similar detections for tomatoes and lettuces have been reported for pesticides such as chlorothalonil, chlorpyrifos, cyfluthrin, γ-cyhalothrin, cypermethrin, and methamidophos in surveillance studies conducted in Xiamen, China [8]. From the total samples evaluated, a high number and concentration of pesticides were detected, mainly in lettuces (Table 2). Several pesticides, such as imidacloprid, γ-cyhalothrin, methamidophos, chlorpyrifos, dithiocarbamates and propamocarb were the main pesticide detected. In the case of imidacloprid, the highest detections were shown in lettuces with a mean of 0.23 mg/kg. Similar results are already reported in the literature. According to a study developed in Turkey, imidacloprid was the most frequent residue found in lettuces with 11 detections. The concentration ranged between 0.03 and 0.46 mg/kg and both values were below their corresponding MRL [26]. However, γ-cyhalothrin showed a mean concentration of 0.14 mg/kg. A sample of lettuce revealed the highest concentration with 11.82 mg/kg of methamidophos, with a mean of 2 mg/kg. The Chilean MRL of methamidophos in lettuce is 0.01 mg/kg. Considering these results, methamidophos represents the highest transgression of Chilean regulation. Supporting our findings, the national surveillance program of Chile reported 13 detections over the MRL of methamidophos for lettuces [12]. Since methamidophos has been banned in several countries, current literature is limited to compare surveillance studies. In our study, a high concentration in two samples of lettuces was detected for difenoconazole with values between 3.45–3.90 mg/kg and a transgression of their Chilean MRLs over 2 mg/kg. In comparison with our results, studies conducted in Valencia, Spain, found γ-cyhalothrin residues in 3% of lettuces with a concentration of 0.07 mg/kg [27]. In a surveillance study in Western China a multiclass of pesticides were investigated in 506 samples of highly consumed types of vegetables including leafy vegetables. From the total samples, 25% contained pesticide residues less than or equal to the MRLs, 4.9% contained residues above the MRL [28].

The results obtained in tomatoes reveal several pesticides, including γ-cyhalothrin, methamidophos, chlorothalonil as the main detections. The highest concentration detected in a sample of tomato was 3 mg/kg of methomyl, while the Chilean MRL is 1 mg/kg. The levels of γ-cyhalothrin detected show a mean of 0.05 mg/kg. The concentrations of methamidophos, chlorothalonil, and acetamiprid were 0.18, 0.85 and 0.49 mg/kg respectively. Similar pesticide residues such as γ-cyhalothrin, acetamiprid, chlorpyrifos, cyfluthrin and chlorothalonil have been determined in greenhouse conditions in the range of 0.02–0.25; 0.02–0.18; 0.01; 0.03; and 0.05–0.06 mg/kg, in tomatoes from Kazakhstan [29]. Studies conducted in Northeast China and Ghana described methamidophos determined in tomatoes with mean concentrations of 0.8 µg/kg [24] and 0.01 ± 0.002 mg/kg respectively [30]. On another hand, concentration values for acetamiprid in tomatoes ranged between 0.01–0.29 mg/kg and 9 transgressions of Turkish MRLs [26].

Table 3 shows a summary of the number of pesticide detections and their compliance with Chilean MRLs. From the total samples evaluated, 56% presented one or multiple residues. The samples with multiple residues show a 17% for tomatoes and 25% for lettuces. In brief, from the samples with pesticides, 52% and 58% have been detected in tomatoes and lettuces, respectively. In addition, 16% (lettuces) and 17% (tomatoes) of samples contained pesticide residues above the Chilean MRLs. In 2017, the NPRMP in Chile evaluated 485 samples of vegetables. From the surveillance, 16.97% of samples were over the Chilean MRLs and 6.9% of samples reported nonauthorized pesticides in different vegetables. From the total samples reported, lettuces and tomatoes show 5.3% (26 samples) and 2.3% (11 samples) over the Chilean MRLs. On another hand, the values for pesticide nonauthorized for lettuces and tomatoes the values were 1.4% (7 samples) and 0.6% (3 samples), respectively [12]. Over the last years, similar results have been presented in surveillance studies conducted in China. Surveillance studies in tomatoes and lettuces reported samples over the Chinese MRLs between 7.5% and 10.4%, respectively [8]. A study conducted in Xinjiang reported pesticide residues in leaf lettuces, lettuces and tomatoes with 7%, 8% and 2% of samples over the MRLs, respectively [15]. However, studies conducted with tomatoes and lettuces in Spain, determined pesticide residues in more than 60% of samples and 8.3% and 4.2% of samples over the European MRLs respectively [31].

### 2.3. Health Risk Assessment

The EDIs were calculated at different age groups using two models. The main difference between the data of MINSAL and WHO was the consumption level for tomatoes and lettuces. The data of WHO used in the model were 10.5 and 2.04 g/day, respectively. However, the consumption levels for the Chilean model were 120 and 50 g/day, respectively. For the health risk assessment, the results obtained with the Chilean model were 11,4- and 24,4-fold higher than the data of WHO. The results in lettuces showed the highest EDIs values in the Chilean model with official data from the Ministry of Health, mainly for the age group 15–24 (Table 4). From the evaluation, five pesticides including cyprodinil, boscalid, difenoconazole, dithiocarbamates and methamidophos showed the highest EDIs in lettuces for both models in all age groups. In the case of cyprodinil, the EDIs were the highest for the Chilean model with values of 4.3, 3.9, 3.8 and 4.1 for the age groups 15–24, 25–44, 44–65 and 65+. For boscalid, the Chilean model revealed higher values with 3.2, 2.9, 2.9 and 3.1 for the age groups 15–24, 25–44, 44–65 and 65+, respectively. For difenoconazole, using the Chilean model, the EDI values were 1.8, 1.6, 1.6 and 1.7 for the age groups 15–24, 25–44, 44–65 and 65+. In addition, the EDI values obtained for dithiocarbamates, methamidophos and fludioxonil were higher or close to that using the Chilean model. Several estimated daily intakes have been reported in surveillance studies. In Xiamen, China, a risk assessment and EDI were conducted in different food items. including lettuces and tomatoes with values 0.0008 µg/kg/bw/day and 0.004 µg/kg/bw/day [8].

The results obtained for tomatoes show the three highest EDIs values for chlorothalonil, methomyl and acetamiprid for the Chilean model, mainly in the age group 15–24. The pesticides described above present the highest EDI values for both models Chile and WHO in all the age group. For methomyl in the Chilean model, EDIs obtained were 5.4, 4.9, 4.8 and 5.2 for the age groups 15–24, 25–44, 44–65 and 65+, respectively. In addition, for chlorothalonil the EDIs values for the Chilean model were 1.50, 1.34, 1.34 and 1.43 for the age groups 15–24, 25–44, and 65+. In an evaluation of vegetables (including lettuces and tomatoes) in a northern metropolis of China, the EDIs showed that the levels of organochlorine pesticide residues were safe after their evaluation [32].

**Figure 1:**
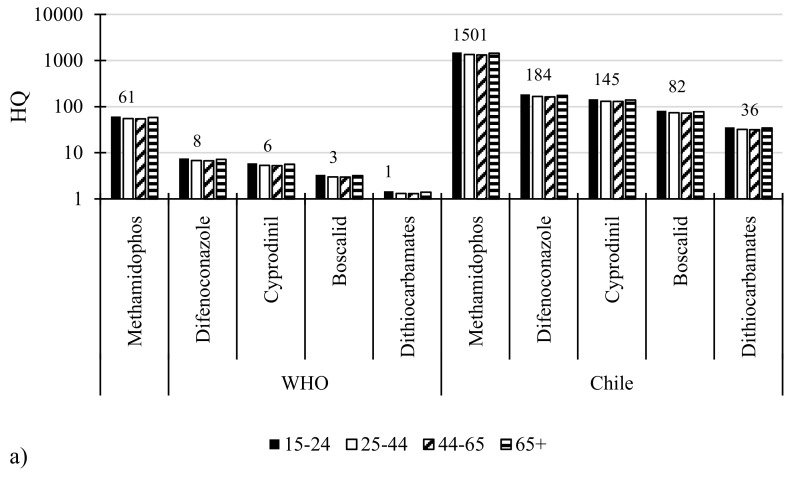

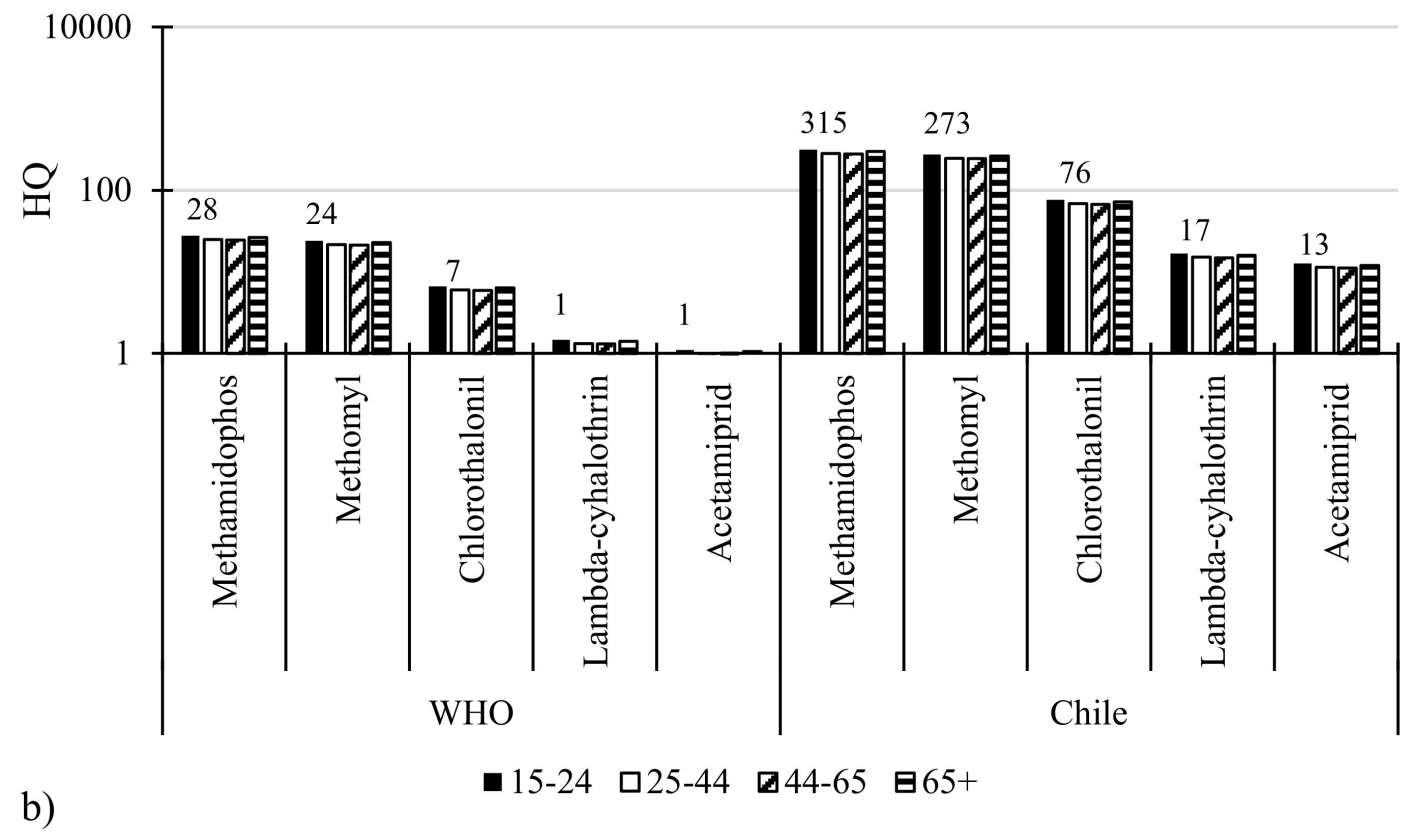
Hazard quotients for tomatoes and lettuces: (**a**) lettuce (**b**) tomato.

The highest hazard quotients (HQs) was the one for methamidophos in all of the evaluations of this study. In general, the main HQs detected in lettuces for pesticides such as methamidophos, difenoconazole, boscalid and cyprodinil (Figure 1a). The highest HQs for all age groups were found for methamidophos with values of 1500, 1348, 1334 and 1435, respectively. In addition, the HQs in the Chilean model described for difenoconazole and cyprodinil in all age groups were higher than 100. The HQs for all pesticides detected decreased in the following order: methamidophos > difenoconazole > cyprodinil > boscalid. The HQs of the pesticides mentioned above represent a current health risk through the consumption of lettuces according to the official data of the Ministry of Health of Chile.

The HQs are described for both models in all age groups. The HQs for all the pesticides residues detected were compared in both models. Vegetables with a HQ greater than 100% are considered potentially unsafe for consumption [33]. For the Chilean model, all the detected HQs were higher than the WHO model.

Supporting these results, the first scientific study of pesticide residues in lettuce, spinach and chard in Chile was conducted in 118 samples at three agricultural areas of Chile. The study concluded that the high levels of methamidophos detected represent a chronic health risk for the Chilean population using the data and model of the World Health Organization [20]. On the other hand, a second study was performed in ready-to-eat leafy vegetables from different supermarkets of the Metropolitana region of Chile. The results showed hazard quotients and hazard indexes in the following order of impact methamidophos > γ-cyhalothrin > chlorpyrifos [21]. Different HQs in lettuces have been reported in surveillances studies. In South Korea, the pesticides residues in leafy vegetables have also been evaluated; their estimated daily intake with HQs lies between 0.0003–30.4% [34].

In the case of tomatoes, the main HQs detected were methamidophos, methomyl and chlorothalonil (Figure 1b). The highest HQs in tomatoes for all age groups were found for methamidophos in the age groups 15–24, 25–44, 44–65 and 65+ with values of 314, 283, 280, and 301, respectively. In addition, the HQs in the Chilean model described for methomyl for all age groups were 273, 246, 243 and 262, respectively. Some studies in tomatoes found chlorothalonil with HQs values: 1.2–1.4 (120–140), 0.6–0.8 (60–80) and 0.4–0.6 (40–60) for children, adolescents and adults, respectively. In the same study HIs were higher than 1 (HI > 100%), for all age groups and doses (maximum value 327), with the exception of adults in the recommended dose (71%) [6]. Comparing the HQs results for adults (chlorothalonil and γ-cyhalothrin), both are in the same range of those obtained for the Chilean model. The potential risk to the consumers through vegetable intake has been estimated by calculating the HQ in unprocessed vegetables from Basque, Spain. In this study, the values obtained ranged between 0.001–0.214% (1–21), demonstrating no health concerns [31].

**Figure 2:**
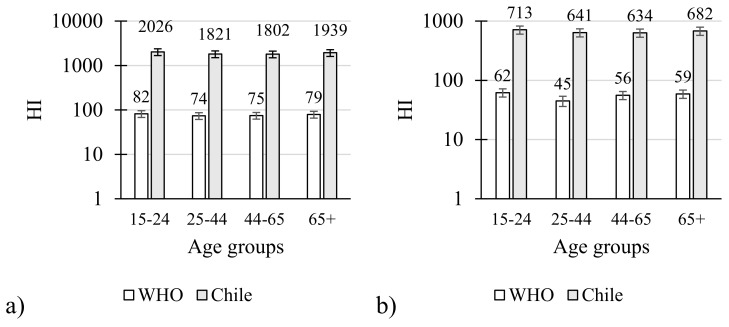
Hazard index for both models in all age groups: (**a**) lettuce; (**b**) tomatoes.

The HQs were summed up in both the Chilean and the WHO model to obtain the hazard index (HI) of pesticide residues. In the case of the Chilean model after summing up all the HQs of each pesticide found in lettuces, the HIs were above 1800 for all age groups (Figure 2a). A similar situation is described for tomatoes with HI values above 500, respectively (Figure 2b). Contrasting results have been reported in the surveillance studies. In general, HI below 1 indicate no risk for consumers. The cumulative dietary exposure of the population of Denmark was evaluated with the HI to carry out a cumulative risk assessment after chronic dietary exposure to pesticides in vegetables, including tomatoes and lettuces for various consumer groups. The HI was below 1 even for consumers who eat more than 550 g of fruit and vegetables per day [35]. In surveillance studies for tomatoes the Hazard Risk Index (HRI) for methamidophos for adults has been described between 1.84 and 1.92. These values were calculated with the Chilean model, representing more than 10-fold higher than the WHO model. Contrasting results have been proposed for methamidophos. In Africa, studies conducted on lettuces and tomatoes showed no risks for the population in Kumasi, Ghana [30]. In the case of residues of methomyl, the HRI value was 0.58, which presents no risks to the exposed population [36]. The hazard index was a measurement of potential risks of adverse health effects from a mixture of pesticide residues in the vegetables evaluated in this study. The high values of HI due to organophosphate insecticides, which are widely used in agriculture, are a cause of acute poisoning and inhibition of neuronyl acetylcholinesterase [24,37].

## 3. Materials and Methods

### 3.1. Study Area

The climate of Metropolitan Region presents hot summers (December-April, up to 30 °C) and short cold winters (May–July, down to 2 °C). The average annual temperature ranges from 12 °C to 15 °C, whereas the average annual rainfall is 312 mm. The rainfall occurs between June and August.

### 3.2. Sampling of Fresh Vegetables

The study was conducted in 2018 in the Metropolitana Region of Chile between August and December. Eighty farms of tomatoes and lettuces were sampled with the assistance of agronomists of the Institute of Agricultural Research (INIA). All the evaluated farmers distribute their products in the Metropolitana Region. The samples were collected at harvest time, including fresh tomatoes (*n* = 23) and lettuces (*n* = 57). The size was a 2-kg of sample wrapped in aluminum foil and transported for analysis. The samples were processed and analysed in the Laboratory of Pesticide Residues and Environmental at INIA Regional Center La Platina. The entire sample was milled and homogenized by a food processor (Oster Company, Boca Raton, FL, USA). Samples were homogenized and 20 g were transferred to 100-mL flasks and stored at −20 °C until the analysis.

### 3.3. Chemicals and Reagents

All of the pesticides we analysed are among the most frequently used in the pest management programs by farmers in Chile and thus, were mostly detected. More than 180 pesticides were evaluated in a preliminary multiresidue screening program. All pesticides are available on the Chilean market and authorized by the Agricultural and Livestock Services of Chile. All chemicals and reagents used in this study were of analytical grade. The analytical-grade pesticide standards (>99% purity) were obtained from Chem Service (West Chester, PA, USA) and Sigma-Aldrich (Saint Louis, MO, USA). All chemicals and reagents (analytical grade) used in this study were obtained from Merck (Darmstadt, Germany) and J.T. Baker (Phillipsburg, NJ, USA). Deionized water was purified with Milli-Q system from Merck (Milford, MA, USA).

### 3.4. Quality Assurance of Method

The quality assurance of method was validated according to the SANTE 11813/2017 guidelines and laboratory procedures of International Organization for Standardization ISO/IEC 17025:2017. The acceptable recovery was defined between 70–120%. The concentration started at 0.01 mg/kg and was further increased depending on the pesticides. In general, the limit of quantification was defined as the lowest concentration with recoveries in the acceptable range. The relative standard deviation was determined by analysing replicate samples at three levels (*n* = 6/level) and was set at <20%.

### 3.5. Pesticide Analysis

The standard method known as the quick, easy, cheap, effective, rugged, and safe (QuEChERS) method was used for the extraction and quantification (prEN 15662) [37,38]. Briefly, two tubes in the QuEChERS system Waters, (Mildford, MA, USA) were used for the extraction and clean-up. The first tube contained 4 g magnesium sulphate (MgSO_4_), 1 g sodium chloride (NaCl), and 1.5 g citrate, and the second tube contained 900 mg MgSO_4_, 150 mg primary secondary amine (PSA), and 150 mg C18. Pesticide groups found in the fresh tomatoes and lettuce were quantified. The organophosphate residues were quantified using an Agilent 7890-Autosampler (Santa Clara, CA, USA) gas chromatography system with a nitrogen-phosphorus detector (NPD) detector. The GC system was equipped with a HP-5MS column from Agilent (Santa Clara, CA, USA). For the halogenated pesticides, a GC-electron capture detector (ECD) Thermo Scientific Trace-Ultra with autosampler (Walthman, MA, USA) and Perkin Elmer Auto-System XL (Walthman, MA, USA) equipped with DB-1 and DB-17 columns from Agilent (Santa Clara, CA, USA). The methyl-carbamates were quantified using a high-performance liquid chromatography (HPLC) system with a Merck Hitachi LaChrom D-7000-autosampler (Dartford, United Kingdom) with a fluorescence (FL) detector and a reaction pump (655A-B) from Merck Hitachi (Darford, United Kingdom). The HPLC was equipped with column waters (Mildford, MA, USA). For the imidacloprid and carbendazim analysis, a HPLC system Merck Hitachi D-6000 with an ultraviolet (UV) detector (Burladingen, Germany) and an Xterra RP-18 column from Waters (Mildford, MA, USA) was used. Before the HPLC analysis, all samples were filtered through a 0.22 µm PVDF syringe filter into an auto sampler vial. The dithiocarbamates levels were determined [39] using distillation and quantification with a Thermo-Genesys 10VIS spectrophotometer (Thermo Scientific Inc., Madison, WI, USA). The results were expressed as milligrams of carbon disulphide per kilogram (mg CS_2_/kg). The values of each pesticide detected were compared with the MRLs in Chile established by the RES-33/2010-02-16 and RES-7627/2011-10-02 based on the MRLs of Codex Alimentarius, and considering MRLs of European Union and USA.

### 3.6. Health Risk Assessment

The results of the monitoring were used to evaluate a health risk assessment and the compliance with Chilean MRLs. The ADIs obtained from the literature are described in the Table 4 [19,20]. The EDI (mg kg/bw/day) was calculated by multiplying the pesticide concentrations of each pesticide (mg/kg) and the food consumption rate (kg/day) and dividing this by the body weight (bw) [40,41]. The body weight data was taken from 2009–2010 Chilean National Health Survey (ENS) conducted in 2009–2010 [42]. Age groups such as 15–24, 25–44, 45–64, 65+ and their corresponding body weights were gathered from the survey and used for the following two scenarios: a) WHO: The food consumption used was based on data from the WHO/Global Environment Monitoring (GEMS)/Food Cluster Diet 2013, a part of the global platform for food safety data and information (FOSCOLLAB). The cluster G05 reports the following consumption level: tomatoes 10.5 g/day and lettuces 2.046 g/day b) Chile: The food consumption used was based on the data provided by the MINSAL. The Chilean consumption for tomatoes was defined as 120 g/day, and lettuce 50 g/day [22]. The consumption spreadsheet was downloaded from the official web page of Ministry of Health of Chile [43]. The long-term risk assessments of the intake compared to the pesticide data were performed by calculating the hazard quotient (HQ = EDI/ADI). Furthermore, an index (%) >100 indicates the possibility that the exposure would induce obvious toxic effects [24]. The HI was calculated with the HQs summed up for all the pesticides considered neurotoxic [40,41]. HI = $\overset{i}{\underset{n=1}{\sum }}HQn$. A HI (%) > 100 indicated that vegetables should be considered a risk to the consumers, whereas an HI (%) < 100 indicated that the consumption was considered acceptable.

## 4. Conclusions

The main pesticides detected in tomatoes and lettuces were methamidophos, methomyl, difenoconazole, cyprodinil and boscalid. The hazard index with the Chilean model revealed the highest risks for all age groups for both vegetables. Our results are similar to the last reports of the national surveillance programs reported in Chile. The necessity to evaluate the health risk assessment is a priority at national level and this methodology should be included in the Chilean regulation to modify the surveillance according to the results obtained. Additional studies are necessary to evaluate specific health risks in different regions of Chile in order to establish their traceability due to lack of information. As soon as the food consumption data in Chile becomes available for children, the Chilean model should include the age group under age 15, because these groups are more vulnerable to a high concentration of pesticide residues. From the results obtained, we strongly suggest to the Agricultural and Livestock Service of Chile reevaluate the registration and commercialization of the pesticides evaluated in this study. The health risk assessment shows strong evidence to support a ban on several pesticides from the Chilean market.

## Figures and Tables

**Table 1:** Quality assurance parameters obtained for the analysis of the target pesticides (*n* = 6).

Pesticide	Analytical Methodology	Limit of Detection (µg/kg)	Limit of Quantification (µg/kg)	% Recovery/RSD Lettuce/Tomato	
Acetamiprid [I]	GC-NPD	5	10	-	116 ± 5.4
Azoxystrobin [F]	GC-ECD	5	10	106 ± 9.7	-
Boscalid [F]	GC-ECD	5	10	102 ± 1.1	97.3 ± 7.4
Chlorfenapyr [I]	GC-ECD	5	10	-	94 ± 3.1
Chlorothalonil [F]	GC-ECD	5	10	72.6 ± 11.9	
Chlorpyrifos [I]	GC-ECD	5	10	93.7 ± 5.2	94.9 ± 6.6
Cyfluthrin [I]	GC-ECD	10	20	92.6 ± 3.1	100.8 ± 3.2
Cypermethrin [I]	GC-ECD	5	10	93.6 ± 8.1	-
Cyprodinil [F]	GC-NPD	5	10	95.5 ± 4.4	-
Difenoconazole [F]	GC-ECD	5	10	99.4 ± 4.2	-
Dimethomorph [F]	GC-ECD	5	10	-	100 ± 3.4
Dithiocarbamates [F]	Colorimetric method	5	10	ND	-
Esfenvalerate [I]	GC-ECD	5	20	89.9 ± 3.7	-
Fludioxonil [F]	GC-NPD	5	10	102.7 ± 4	-
Imidacloprid [I]	HPLC-DAD	10	20	94.7 ± 14	100.5 ± 10.2
Iprodione [F]	GC-ECD	5	10	101 ± 2.8	103.4 ± 2.2
γ-cyhalothrin [I]	GC-ECD	5	10	99.1 ± 3.2	100.2 ± 9.2
Metalaxyl [F]	GC-NPD	5	10	96.1 ± 2.2	-
Methamidophos [I]	GC-NPD	5	10	85 ± 4.1	108 ± 10.6
Methomyl [I]	HPLC-FL	5	10	101.1 ± 9.8	93.5 ± 10.9
Propamocarb [F]	GC-NPD	5	10	-	75 ± 6.4

[I] = Insecticide; [F] = Fungicides; ND = not determined.

**Table 2:** Pesticides detections in tomatoes (*n* = 23) and lettuces (*n* = 57).

Vegetable	Pesticide	Median	Max Value (mg/kg)	Mean	MRL
**Lettuce**	Azoxystrobin	5	5	0.28 ± 0.09	5
	Boscalid	4.1	8.73	4.37 ± 0.2	10
	Chlorothalonil	-	0.61	0.61	0.01
	Chlorpyrifos	1	1	0.08 ± 0.11	0.1
	Cyfluthrin	-	0.07	0.07	3
	Cypermethrin	-	0.02	0.02	0.7
	Cyprodinil	-	5.82	5.82	10
	Difenoconazole	2	2	2.46 ± 2.12	2
	Dithiocarbamates	10	10	2.39 ± 3.74	10
	Esfenvalerate	0.02	0.02	0.02 ± 0.01	0.02
	Fludioxonil	10	2.5	1.38 ± 1.58	10
	Imidacloprid	3.5	3.5	0.23 ± 0.45	3.5
	Iprodione	-	1.32	1.32	25
	γ-cyhalothrin	2	2	0.14 ± 0.2	2
	Metalaxyl	5	5	0.07 ± 0.06	5
	Methamidophos	0.01	0.01	2 ± 5.26	0.01
	Methomyl	0.2	0.2	0.04 ± 0.02	0.2
	Propamocarb	100	100	0.43 ± 0.82	100
**Tomato**	Acetamiprid	0.2	0.89	0.49 ± 0.57	0.2
	Boscalid	-	-	<LOQ	1.2
	Chlorfenapyr	-	-	<LOQ	1
	Chlorothalonil	5	1.64	0.85 ± 1.12	5
	Chlorpyrifos	-	0.02	0.02	0.5
	Cyfluthrin	-	0.04	0.04	0.2
	Dimethomorph	-	0.03	0.03	1
	Imidacloprid	-	0.27	0.27	0.5
	Iprodione	-	0.3	0.16	5
	γ-cyhalothrin	0.05	0.1	0.05 ± 0.07	0.3
	Methamidophos	0.01	0.33	0.18 ± 0.22	0.01
	Methomyl	0.02	1	3.04 ± 0.01	1

**Table 3:** The pesticide residues detected in vegetables.

Vegetable	Samples	Samples Free Residues		No of Samples ≻ MRL		Samples with Multiple Residues	
Type	N^o^	N^o^	%	N^o^	%	N^o^	%
Lettuce	57	24	42	9	16	14	25
Tomato	23	11	48	4	17	4	17
Total samples	80	35	44	13	16	18	23

**Table 4:** The Estimated Daily Intake (mg kg/bw/day) for lettuces and tomatoes.

Pesticides	Acceptable Daily Intake (ADI) (mg/kg)	a) EDI from WHO				b) EDI from Chile			
		15–24	25–44	44–65	65+	15–24	25–44	44–65	65+
**Lettuces**									
Imidacloprid	0.06	0.007	0.006	0.006	0.007	0.174	0.156	0.154	0.166
γ-cyhalothrin	0.005	0.004	0.004	0.004	0.004	0.104	0.094	0.093	0.1
Methamidophos	0.001	0.061	0.055	0.055	0.059	1.501	1.3	1.3	1.436
Chlorpyrifos	0.01	0.002	0.002	0.002	0.002	0.061	0.055	0.054	0.058
Dithiocarbamates	0.05	0.073	0.066	0.065	0.07	1.792	1.6	1.5	1.714
Propamocarb	0.4	0.013	0.012	0.012	0.013	0.323	0.29	0.287	0.309
Difenoconazole	0.01	0.075	0.068	0.067	0.072	1.8	1.6	1.6	1.7
Esfenvalerate	0.02	0.001	0.001	0.001	0.001	0.019	0.017	0.017	0.018
Azoxystrobin	0.2	0.009	0.008	0.008	0.008	0.211	0.19	0.188	0.202
Boscalid	0.04	0.134	0.12	0.119	0.128	3.2	2.9	2.9	3.1
Chlorothalonil	0.02	0.019	0.017	0.017	0.018	0.456	0.41	0.405	0.436
Methomyl	0.02	0.001	0.001	0.001	0.001	0.033	0.03	0.03	0.032
Cyfluthrin	0.04	0.002	0.002	0.002	0.002	0.052	0.047	0.047	0.05
Cyprodinil	0.03	0.179	0.16	0.159	0.171	4.3	3.9	3.8	4.1
Fludioxonil	0.4	0.042	0.038	0.038	0.04	1	0.929	0.919	0.989
Iprodione	0.06	0.04	0.036	0.036	0.039	0.99	0.889	0.88	0.947
Metalaxyl	0.08	0.002	0.002	0.002	0.002	0.055	0.049	0.049	0.052
Cypermethrin	0.02	0.001	0.001	0.001	0.001	0.015	0.013	0.013	0.014
**Tomatoes**									
Imidacloprid	0.06	0.043	0.038	0.038	0.041	0.486	0.437	0.432	0.465
γ-cyhalothrin	0.005	0.007	0.007	0.007	0.007	0.084	0.075	0.075	0.08
Methamidophos	0.001	0.028	0.025	0.025	0.026	0.315	0.283	0.28	0.301
Chlorpyrifos	0.01	0.003	0.003	0.003	0.003	0.036	0.032	0.032	0.034
Boscalid	0.04	0.002	0.001	0.001	0.002	0.018	0.016	0.016	0.017
Chlorothalonil	0.02	0.133	0.12	0.118	0.127	1.5	1.3	1.3	1.4
Methomyl	0.02	0.479	0.43	0.426	0.458	5.4	4.9	4.8	5.2
Acetamiprid	0.07	0.077	0.069	0.069	0.074	0.882	0.792	0.784	0.844
Cyfluthrin	0.04	0.006	0.006	0.006	0.006	0.072	0.065	0.064	0.069
Iprodione	0.04	0.025	0.023	0.022	0.024	0.288	0.259	0.256	0.275
Chlorfenapyr	0.03	0.002	0.001	0.001	0.002	0.018	0.016	0.016	0.017
Dimethomorph	0.2	0.005	0.004	0.004	0.005	0.054	0.049	0.048	0.052
